# Autochthonous Human *Dirofilaria repens* Infection in Austria

**DOI:** 10.1007/s11686-021-00506-0

**Published:** 2022-01-12

**Authors:** Nora Geissler, Johanna Ruff, Julia Walochnik, Wilhelm Ludwig, Herbert Auer, Ursula Wiedermann, Werner Geissler

**Affiliations:** 1 Institute of Specific Prophylaxis and Tropical Medicine, Medical University of Vienna, Vienna, Austria; 2 Private Doctor’s Office, Vienna, Austria

**Keywords:** Dirofilariasis, Nematode infections, Parasitic worms, Climate change, Austria

## Abstract

**Purpose:**

This report describes a rare autochthonous case of human *D. repens* infection in Austria. Dirofilariosis is a mosquito-borne parasitic infection that predominantly affects dogs. Human *D. repens* infections have primarily been reported in Mediterranean countries, but are emerging throughout Central and Northern Europe.

**Methods:**

The worm was removed surgically and identified using PCR and DNA sequencing. The consensus sequences were compared against reference sequences of *Dirofilaria repens* from GenBank.

**Results:**

The 56-year-old woman acquired the infection, which presented as a subcutaneous nodule, in Vienna, Austria. This is the second autochthonous case of human *D. repens* infection in Austria.

**Conclusion:**

The reasons for the emergence of *D. repens* and other parasitic infections in Central and Northern Europe are manifold, including climate change and globalization. This case demonstrates that with the growing number of *D. repens* infections*,* health care professionals must place further emphasis on emerging infectious diseases to ensure appropriate diagnostics and treatment in the future.

## Introduction

*Dirofilaria repens* is a vector-borne nematode transmitted by different mosquitoes species. The natural hosts for *D. repens* are dogs, but the parasite can also infest other species, including cats, foxes, and occasionally humans [1]. Since parasite development within the vector is temperature-dependent, the prevalence of *D. repens* in Europe is highest in Mediterranean countries [2–6]. In addition, other Central and Eastern European countries, such as Slovakia, Romania and Hungary, are considered to be endemic for *D. repens* [3, 7–11]. In recent years, however, the number of cases in dogs and humans has increased throughout Central and Northern Europe [12–14]. Adult worms typically inhabit subcutaneous or ocular tissues. Humans are only accidental hosts for *D. repens* nematodes as they usually do not develop to the fertile adult stage. However, (im)mature worms can cause the formation of skin nodules [15] composed of granulomatous tissue, which can be surgically removed [3]. In Austria, several cases of human dirofilariosis have been reported, primarily associated with traveling to endemic areas [1]. In 2008, the first and, until now, only autochthonous case reported in Austria was a patient from a village close to the Hungarian border in the federal state of Burgenland [1, 16].

## Case Report

**Fig. 1:**
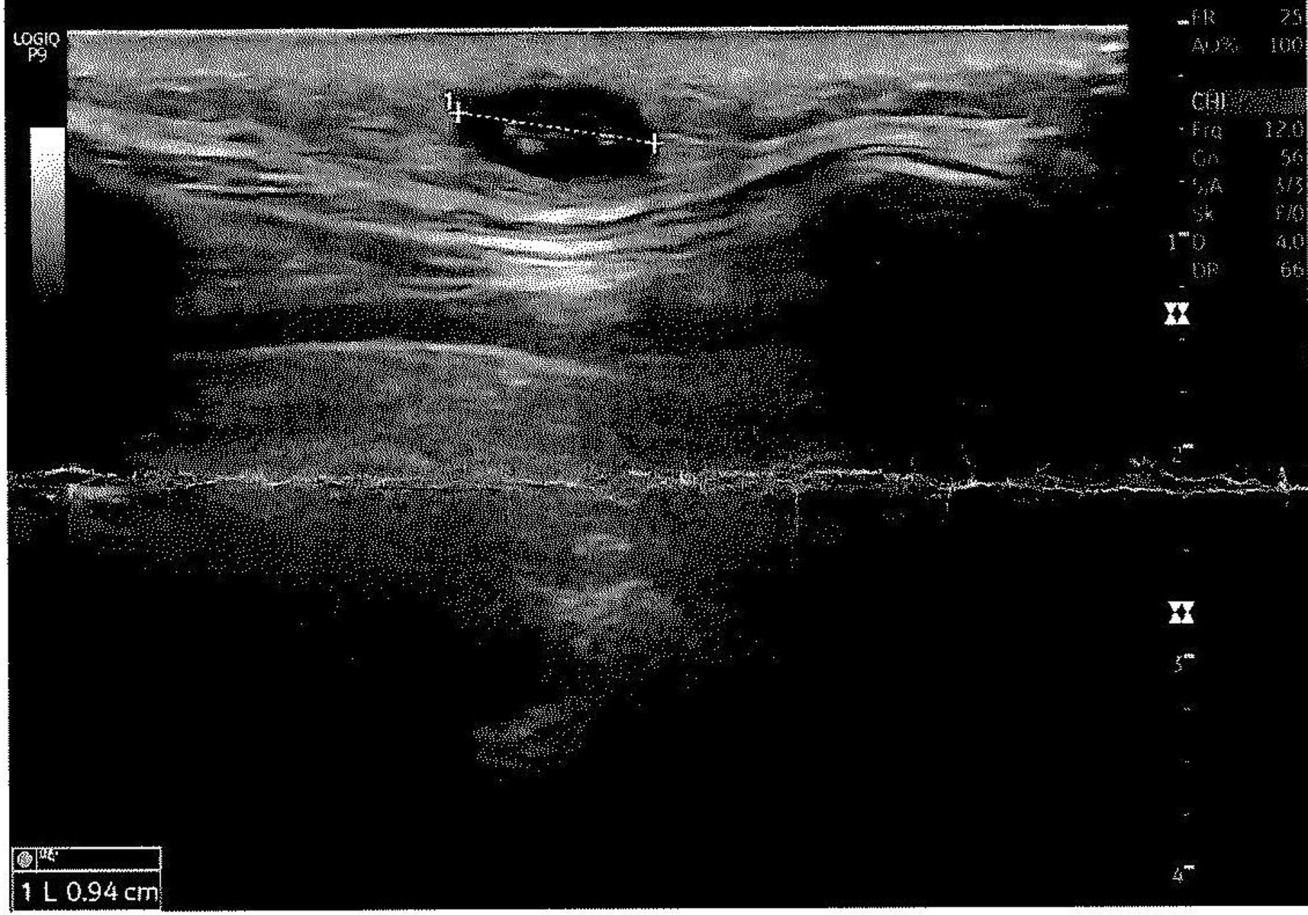
Ultrasound image showing a subcutaneous, hypoechoic, oval, 0.9 cm nodule with a central linear mass of 0.5 cm

**Fig. 2:**
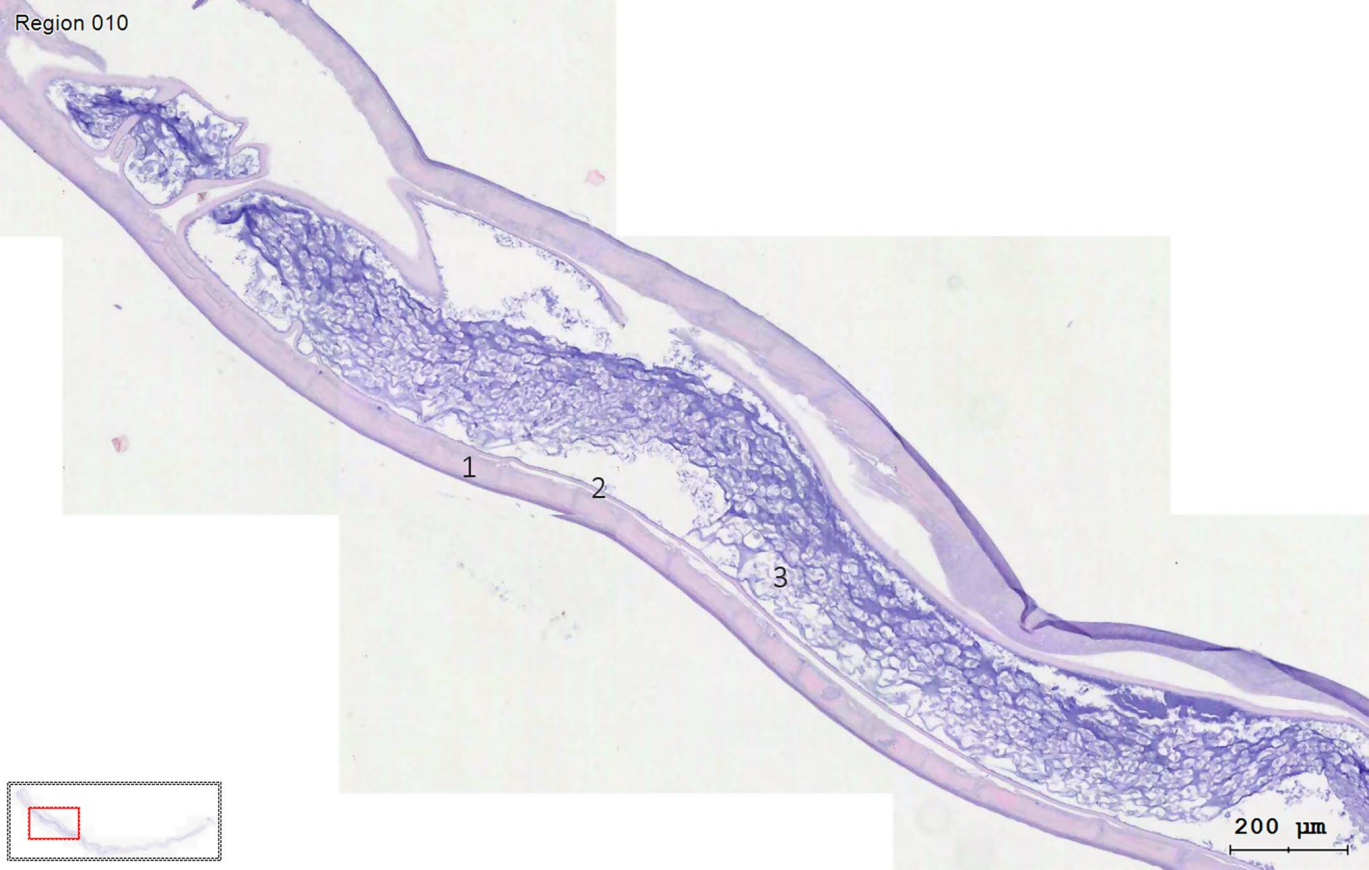
Picture of a longitudinal hematoxylin–eosin-stained histological section of the paraffin-embedded *D. repens* taken using a TissueFAXS microscope ×200 magnification and TissueFAXS software from Tissue Gnostic. In the picture, a thick multilayered cuticle (**1**), the muscle layer (**2**), and the intestinal tract (**3**) are visible

In October 2020, a 56-year-old female patient from Vienna, Austria, presented to her surgeon with 3–4 months’ history of an itching nodule on her left lower costal arch. Other symptoms, such as fever, were denied. The patient was otherwise healthy with no relevant medical history. She reported being bitten by a mosquito while swimming in the Lobau, a floodplain area in Vienna, Austria, 3–4 months ago. The site of the mosquito bite remained itchy and reddish for a week until dissolving spontaneously. 2 to 3 weeks later, the patient noticed a small, slowly growing nodule at the site of the bite. Since then, the swelling continued to grow and cause itching sensations. The patient denied recent travel. Her previous vacation abroad was 2 years ago in Croatia. The woman had a pet dog, which was reported to be healthy. By physical examination, a 2 × 3 cm smooth swelling, painless on palpation, was detected, at the side of the left costal arch region. The skin was intact without signs of inflammation. There was no enlargement of lymph nodes or other pathological findings. Laboratory parameters were unremarkable with eosinophils within the normal range (118/mm^3^). Sonography revealed a subcutaneous, hypoechoic 0.9 × 0.5 cm swelling without signs of inflammation (Fig. 1). A surgical excision was performed under local anesthesia. It revealed a 10 cm, white, slim, worm-shaped organism, which was first sent to a local laboratory (Fig. 2) and subsequently to the national reference laboratory for identification. After morphological investigation, the worm was subjected to molecular analysis. An approximately 0.5-cm-long worm fragment was cut into small pieces using a sterile scalpel and homogenized using a Precellys^®^ bead beater (VWR, Vienna, Austria). Whole-cell DNA was isolated using the QIAamp DNA Mini Kit (Qiagen, Hildesheim, Germany), following the manufacturer’s instructions for tissue samples. First, a polymerase chain reaction specific to *D. repens* [17] was performed and showed a positive result. For further investigation, the cytochrome oxidase (Cox I) gene was amplified by PCR using the universal primers described by Folmer *et al*. [18]. The diagnosis was confirmed by amplifying the internal transcribed spacer 1 (ITS1) of the ribosomal DNA using the pan-filarial primers described by Koehsler *et al*. [19]. The obtained amplicons were extracted from the agarose gels using the QIAquick^®^ Gel Extraction Kit (Qiagen) and subjected to DNA sequencing. Sequences were obtained from both strands in two independent setups by direct sequencing using an automated ABI PRISM 310 Sequencer (PE Applied Biosystems, Langen, Germany) and assembled to consensus sequences using GeneDoc [20]. All consensus sequences were compared against reference sequences of *Dirofilaria repens* available at GenBank by BLAST [21]. Sequence data obtained in this study was submitted to GenBank and is available under the following accession numbers: OL614756 (CoxI) and OL616131 (ITS1).

## Discussion

This paper presents the second autochthonous dirofilariosis caused by *D. repens* in Austria. In 1978, the first case of human dirofilariosis was detected in Austria. The patient probably acquired the infection during a vacation in Greece [22]. Since then, numbers of human *Dirofilaria* cases in Austria have increased steadily. A review of dirofilariosis in Austria between 1978 and 2020 reported 39 human *Dirofilaria* cases. The highest incidence of *Dirofilaria* infection was collected between 2010 and 2019 (15 cases). Of the reviewed dirofilariosis cases, 89.7% were identified as *D. repens* infections [23]. The first autochthonous case occurred in 2008 when a 34-year-old woman was bitten by a mosquito at the Austrian–Hungarian border [16]. Other *Dirofilaria* infections in Austria have been associated with a travel history to endemic regions, especially Mediterranean countries and Hungary [1, 23–25]. Between 1978 and 2018, 47 of *D. repens* cases were documented in Austrian dogs, most of which had a travel history to Hungary or the Western Balkans. However, eight of the infected dogs had no travel history outside Austria [1, 26]. Due to the worm’s subcutaneous location and the often-complete absence of clinical signs in animal hosts, the actual number is expected to be higher. *D. repens* can also infect other wild animals, such as foxes. To date, however, no foxes have tested positive in Austria. Ultimately, wild animal hosts play a minor role in spreading the parasite in Central Europe. According to data available to date, the main factor influencing the emergence of *D. repens* in Austria appears to be the importation of infected dogs and travel with dogs to endemic countries. Therefore, to control the spread of *D. repens* infections in Austria, it is essential to improve diagnosis numbers and refine therapy and infection prevention measures in the canine reservoir. In Austria, several mosquito species can serve as competent vectors for *D. repens*. In October 2012, the first autochthonous cases of mosquitoes infected with *D. repens* were described in *Anopheles maculipennis* and *A. algeriensis* pools in the eastern federal state of Burgenland [27]. Thus, the first autochthonous human infection and the first findings in mosquitoes were reported in a region close to the Hungarian border, suggesting the dispersal of infected mosquitoes into eastern Austria from Hungary. The patient in the presented case study reported having been bitten by a mosquito in the Lobau, a floodplain area in the southeast of Vienna, also in the relative vicinity of the Slovakian and Hungarian borders. A climatic change toward warmer temperatures will eventually contribute to increased cases of dirofilariosis in the future. A higher annual mean temperature accelerates the development of *D. repens* within the vector [28]. Sassnau *et al*. confirmed that by 2014, many regions of Germany had already reached a periodically suitable climate for *D. repens* microfilariae to develop into infectious larvae within the vector [29]. Therefore, regular screening for *D. repens* in mosquitoes should be implemented to keep track of the dispersal. After surgical excision, the worm in this case study was initially sent to a local diagnostic laboratory in the state of Lower Austria, where a definite identification could not be made. It was thus transferred to the national reference laboratory under the preliminary diagnosis of *A. lumbricoides*. Here, it was identified as *D. repens*. *A. lumbricoides* is a nematode that develops in the small intestine of humans and is transmitted by the ingestion of embryonized eggs [30].The morphology of *A. lumbricoides* as well as its life cycle is quite distinct from *D. repens*. However, due to the low incidence of nematode infections in Austria, routine laboratories might have a lack of knowledge on how to correctly diagnose them. The rising incidence of *D. repens* cases in Central and Northern Europe [31] underlines the importance of well-established collaborations between routine and reference laboratories. Finally, it appears essential to enhance training and regular skills among health care professionals to improve diagnostics and the treatment of parasitic and other emerging infections [32, 33].

## Data Availability

Data available on request due to privacy/ethical restrictions.
